# Social support, resource utilization, and well-being: a survey of adolescent parents in Washington, DC

**DOI:** 10.1186/s12889-023-16185-7

**Published:** 2023-07-03

**Authors:** Yael Smiley, Jaytoya Manget, John Barber, Iana Clarence, Nkechi Enwerem, Christiana Jolda, Patricia Quinn, Zillah Jackson Wesley, Davene White, Binny Chokshi

**Affiliations:** 1 Department of General and Community Pediatrics, Children’s National Hospital, 111 Michigan Ave, Washington, DC 20010 USA; 2 Department of Biostatistics and Study Methodology, Children’s National Hospital, Washington, DC USA; 3 District of Columbia Primary Care Association, Washington, DC USA; 4 Division of Nursing, College of Nursing and Allied Health Sciences, Howard University, Washington, DC USA; 5 Department of Pediatrics, Division of Military Child and Family Research, Uniformed Services University of the Health Sciences, Bethesda, MD USA

**Keywords:** Adolescent parents, Well-being, Social support

## Abstract

**Background:**

Adolescent parents experience worse health and socioeconomic outcomes compared to older parents. Little is known about the factors that can lead to better health and well-being among teen-headed families. A city-wide collaborative conducted a comprehensive well-being assessment of expectant and parenting teens in Washington, DC.

**Methods:**

An online, anonymous survey was conducted with adolescent parents in Washington, DC, using convenience sampling. The survey consisted of 66 questions adapted from validated scales of quality of life and well-being. Descriptive statistics were used to describe the data overall, by subgroups of mother and father, and by subgroups of parent age. Spearman’s correlations were utilized to demonstrate associations of social supports with well-being metrics.

**Results:**

A total of 107 adolescent and young adult parents from Washington, DC, completed the survey; 80% of respondents identified as mothers and 20% as fathers. Younger adolescent parents rated their physical health better compared to older adolescent and young adult parents. Adolescent parents reported accessing various governmental and community-based resources in the preceding 6 months. The most used resources were supplemental food programs, with 35% receiving Supplemental Nutrition Assistance Program benefits and 24% receiving support from the Special Supplemental Nutrition Program for Women, Infants and Children. There was no significant difference in health-related well-being metrics among those who did and did not receive resources. Having higher self-reported social support was positively correlated with higher self-rated physical health, mental health, and well-being, as well as experiencing positive emotions, and was negatively correlated with experiencing negative emotions.

**Conclusion:**

This snapshot of the well-being of expectant and parenting teens in Washington, DC, showed overall positive physical, mental, and emotional health. Greater social support was correlated with better outcomes in these areas. Future work will leverage the multidisciplinary collaborative to translate these findings into policies and programs that meet the needs of this population.

## Background

Adolescent pregnancy and parenting is an international issue, and globally 18% of births occur to mothers under twenty years old [1]. In the United States, the teen birth rate has fallen each year since 2009 and in 2019 was 16.7 births per 1,000 [2], however the rate remains higher than that in most developed countries [3]. Over 170,000 adolescents in the United States became parents in 2019 [2]. Expectant and parenting youth face a host of health and socioeconomic challenges. Adolescent parents are more likely to rely on public assistance [4] and are less likely to finish high school [5] compared to their peers. Parenting teens are at high risk for sexually transmitted infections [6] and rapid repeat teen pregnancy, which nearly triples the risk for preterm delivery and stillbirth [7]. They are also more likely to be involved in foster care and the juvenile justice system than their nonparenting peers [8]. Adolescent mothers experience significantly higher rates of depression and other mental health disorders than their nonpregnant peers [9, 10], along with inconsistent engagement in health care services [11, 12]. With support from federal funding streams, programs in healthcare, school, and community-based settings have been developed to address these challenges and improve health and socioeconomic outcomes for adolescent parents and their children [13]. Some programs have achieved measurable reductions in repeat teen pregnancies [14] and improvements in high school graduation rates [15]. Little is known about the factors that can lead to better health and well-being among teen-headed families.

### Protective factors among expectant and parenting adolescents

Although the published literature emphasizes the risks and negative outcomes for teen parents and their children [4], there are likely unstudied protective factors that can mitigate negative outcomes. For example, small studies have identified the importance of social support among teen parents. A teen parent support group in Central Texas called “Mama Club” surveyed characteristics and attitudes of participants [16]. “Socializing with others” and “making friends” were among the factors of the greatest importance to mothers who attended Mama Club, with participants rating these elements as more important than receiving diapers, clothes, and meals [16]. Another US study found that among adolescent parents at 6 months postpartum, greater social support was associated with increased parenting self-efficacy [17]. Attending a support group and experiencing increased self-efficacy likely confer benefits to teen parents, but these studies did not measure a direct link between social support and health and well-being factors. Further investigation into the protective factors for adolescent parents is warranted to improve outcomes for this population.

### The Washington, DC, context

There are approximately 300 teen births in Washington, DC, each year [18]. Among these, the rate of births to younger teens 15 to 17 years old is nearly double the national average [2]. Compared to national averages, youth in the District of Columbia are more likely to have ever had sexual intercourse, have four or more sexual partners, and initiated sex before the age of 13 [19]. Importantly, DC youth were nearly twice as likely to report using no method to prevent pregnancy during their last sexual intercourse compared to the national average [19]. To improve the health and well-being of expectant and parenting youth in Washington, DC, the District of Columbia Network for Expectant and Parenting Teens, or “DC NEXT!” was founded in 2020. The collaboration is a collective impact innovation network funded by a 3-year grant awarded by the Office of Population Affairs [20]. It relies on and employs a human-centered design approach, bringing the voices and perspectives of expectant and teen parents to develop and launch innovations that will improve the well-being of young families in the District of Columbia.

DC NEXT! conducted a survey to understand demographics and well-being factors among expectant and parenting teens in Washington, DC. Well-being assessments evaluate mental health and physical health along with domains such as use of community resources, social health, and financial, food, and housing security [21]. Well-being assessments can measure self-perceived health status, adoption of healthy behaviors, personal productivity, and factors in the physical and social environment that support health [6, 22]. The objective of the study was to understand demographic and well-being factors among teen parents in Washington DC.

## Methods

### Study design

The DC NEXT! Leadership Team created a survey consisting of 66 questions related to expectant and parenting teen well-being. The domains included basic demographics, impact of support systems, views on parenting and stressors, resource utilization, and self-reported assessment of physical and mental health, including the presence of positive and negative emotions. Participants were offered the chance to receive a $25 gift card as an incentive for taking the survey. Convenience sampling was used and survey responses were reviewed before analysis to ensure that responses met inclusion criteria. The Children’s National Hospital Institutional Review Board approved the study.

### Survey instrument

The survey questions related to health and well-being were adapted from validated scales of quality of life and well-being, including the Short Form 36 (SF-36) Health Survey, the RAND Corporation Social Support Survey [22], and the Well-Being in the Nation survey [21]. The questions were scored by respondents on a Likert scale that correlated with negative or positive views of their health and well-being (from poor to very good). Participants were asked to rank their financial situation, educational goals, current self-perception, and projected standing in 5 years’ time on the Cantril ladder [23]. The ladder is numbered from 0 to 10, with the top representing the best possible life for you and the bottom representing the worst possible life [24, 25]. To assess use of community resources, a list of potential resources was developed by the study team who have knowledge of the resources available to teen parents in the region. Respondents were asked to select all resources they had utilized in the preceding six months. The survey was pilot tested by a group of teen parents to ensure clarity of questions.

### Data collection

An anonymous, online, public survey link was created in REDCap and distributed to DC NEXT! network partners who provide services to teen parents. Network partners publicized the survey using social media, email, and direct outreach to individuals meeting the inclusion criteria of living in the DC area, being 21 years or younger and becoming a parent by age 19, or being 19 years or younger and being pregnant at the time of taking the survey. Both adolescent mothers and fathers were eligible to take the survey.

### Statistical analysis

All surveys were reviewed prior to statistical analysis, and surveys were excluded that were incomplete or did not meet inclusion criteria based on age of respondent. Descriptive statistics were used to describe the data overall, by subgroups of parental identification (mother/father), and by subgroups of parent age. Continuous data were presented with mean and standard deviation, whereas categorical data were presented with count and frequency. Subgroup analyses were conducted to examine statistical differences in health and well-being outcomes between groups of parental identification (mother/father) and by subgroups of parent age. To analyze resource utilization, each resource received in the preceding six months was tallied and frequency calculated as a percentage of total respondents. Participants were divided into two subgroups of “1 or more resource used” and “no resources used” and a subgroup analysis comparing service utilization and well-being was assessed. The variable 1 or more service was created based on the prevailing distribution of receipt of services and was preferable to a heavily skewed continuous alternative. Spearman’s correlations were utilized to demonstrate associations of social supports with well-being metrics. All 2 group comparisons were performed using the Wilcoxon Rank Sum Test and comparisons involving more than 2 groups were performed with the Kruskal Wallis Test. Analyses were performed in SAS V9.4 (Cary, NC) with *P* < 0.05 deemed statistically significant.

## Results

A total of 230 survey responses were received. Responses were excluded that were incomplete (56) or did not meet inclusion criteria based on age (67). 107 adolescent and young adult parents from Washington, DC, completed the survey (Table 1). Most respondents identified as mothers (80%), Black/African American (86%), and straight (72%). Age-specific demographics showed that 22% of respondents were 17 years or younger, 40% were older adolescent parents (18–19 years), and 37% were young adult parents (20–21 years). About one-third of the overall respondents were in school, and nearly half were employed. Of those who were not employed, the majority (35%) said they were looking for work.

**Table 1:** Demographic results overall and by subgroups

	Mean (standard deviation) or frequency (%)					
	Overall	Subgroup: Parent Type		Subgroup: Age (years)		
		Mother	Father	≤ 17	18–19	20–21
N	107	86 (80%)	21 (20%)	24 (22%)	43 (40%)	40 (37%)
Age (years)	19 (2)	19 (2)	19 (1)	15 (1)	19 (0)	20 (1)
Race category						
American Indian or Alaskan Native	2 (2%)	1 (2%)	1 (5%)	1 (4%)	1 (2%)	0 (0%)
Black or African American	92 (86%)	76 (88%)	16 (76%)	19 (79%)	37 (86%)	36 (90%)
Native Hawaiian/Other Pacific Islander	1 (1%)	1 (2%)	0 (0%)	0 (0%)	1 (2%)	0 (0%)
White	4 (4%)	1 (2%)	3 (14%)	0 (0%)	2 (5%)	2 (5%)
Some other race or origin	4 (4%)	3 (3%)	1 (5%)	1 (4%)	1 (2%)	2 (5%)
I prefer not to respond	4 (4%)	4 (5%)	0 (0%)	3 (13%)	1 (2%)	0 (0%)
Hispanic category						
No, not of Hispanic, Latino/a/x, or Spanish origin	91 (85%)	74 (86%)	17 (81%)	18 (75%)	36 (84%)	37 (93%)
Yes, of Hispanic, Latino/a/x, or of Spanish origin	12 (11%)	8 (9%)	4 (19%)	2 (8%)	7 (16%)	3 (8%)
I prefer not to respond	4 (4%)	4 (5%)	0 (0%)	4 (17%)	0 (0%)	0 (0%)
Sexual identity						
Asexual	5 (5%)	4 (5%)	1 (5%)	1 (4%)	2 (5%)	2 (5%)
Bisexual	12 (11%)	11 (13%)	1 (5%)	4 (17%)	6 (14%)	2 (5%)
Demisexual	2 (2%)	2 (2%)	0 (0%)	0 (0%)	2 (5%)	0 (0%)
Gay	3 (3%)	2 (2%)	1 (5%)	0 (0%)	1 (2%)	2 (5%)
Lesbian	1 (1%)	1 (1%)	0 (0%)	0 (0%)	0 (0%)	1 (3%)
Pansexual	2 (2%)	2 (2%)	0 (0%)	1 (4%)	0 (0%)	1 (3%)
Straight, that is, not lesbian or gay	77 (72%)	59 (69%)	18 (86%)	14 (58%)	31 (72%)	32 (80%)
I prefer not to respond	5 (5%)	5 (6%)	0 (0%)	4 (17%)	1 (2%)	0 (0%)
Currently in school	39 (36%)	34 (40%)	5 (24%)	21 (88%)	12 (28%)	6 (15%)
Currently employed	46 (43%)	35 (41%)	11 (52%)	3 (13%)	21 (49%)	18 (45%)
Physical health (5-point scale)	4.1 (0.9)	4.1 (0.9)	4.1 (1.0)	4.7 (0.6)*	3.9 (1.0)	4.1 (1.0)
Mental health (5-point scale)	3.7 (1.1)	3.7 (1.1)	3.8 (1.0)	3.8 (1.3)	3.7 (1.0)	3.7 (1.1)
Negative emotions (6-point scale)	2.5 (1.4)	2.5 (1.4)	2.5 (1.5)	2.6 (1.9)	2.4 (1.1)	2.6 (1.4)
Positive emotions (6-point scale)	4.7 (1.3)	4.7 (1.3)	4.5 (1.2)	5.0 (1.5)	4.6 (1.2)	4.5 (1.2)
Cantril ladder score (10-point scale)	6.2 (2.2)	6.1 (2.2)	6.4 (2.3)	6.6 (2.0)	6.3 (2.6)	5.8 (1.9)

* *p* < 0.05

Adolescent parents reported accessing various governmental and community-based resources in the preceding 6 months (Table 2). The most used resources were supplemental food programs, with 35% receiving Supplemental Nutrition Assistance Program (SNAP) benefits and 24% receiving support from the Special Supplemental Nutrition Program for Women, Infants and Children (WIC), followed by food pantries, Temporary Assistance for Needy Families (TANF), housing assistance, and childcare. However, the largest group of respondents, 37%, reported receiving no resources.

**Table 2:** Resources received in the past 6 months

Resources received (all that apply)	Frequency (%)
Child care	11 (10%)
Food pantry	18 (17%)
Greater DC diaper bank	8 (7%)
Housing assistance (e.g., subsidized housing)	12 (11%)
SNAP (Supplemental Nutrition Assistance Program/food stamps)	37 (35%)
TANF (Temporary Assistance for Needy Families)	17 (16%)
Unemployment Insurance	1 (1%)
WIC (Women, Infants, Children Program)	26 (24%)
Other aid from the government	3 (3%)
None	40 (37%)

Descriptive statistics highlighted differences between subgroups of teen parents were (Table 1). Fathers had higher rates of straight sexual identity (86%) and current employment (52%) compared to mothers. Only 24% of fathers were currently in school and the majority, 71%, reported accessing no resources. Younger adolescents were less likely to report straight sexual identity (58%), compared to 72% of older adolescent parents and 80% of young adult parents overall. Younger adolescents had higher rates of being in school (88%) and lower rates of being employed (13%) compared to older adolescent and young adult parents.

Of the well-being metrics that were analyzed, mean self-reported scores from all respondents measured 4.1/5 for physical health and 3.7/5 for mental health. The overall Cantril ladder score was 6.2/10, which signifies moderate to high overall well-being [24, 25]. As shown in Table 1, in the subgroup analysis there were no statistically significant differences between mothers and fathers in ratings of physical health, mental health, presence of positive or negative emotions, or Cantril ladder score (P > 0.05 for all comparisons). Younger adolescent parents rated their physical health better than older adolescent and young adult parents, and this difference was statistically significant (Table 1). There was no statistically significant difference in health-related well-being metrics among those who did and did not receive resources.

Five health and well-being metrics were correlated with questions measuring social support (Table 3). Spearman’s correlations ranged from − 0.31 to 0.39. Having higher self-reported social support was positively correlated with higher self-rated physical health, mental health, and well-being and with experiencing positive emotions and was negatively correlated with experiencing negative emotions.

**Table 3:** Correlations between social supports and health and well-being metrics

Social supports	Health and well-being metrics				
	Physical health	Mental health	Positive emotions	Negative emotions	Cantril ladder
Have family or friends you could count on to help you when you needed them	0.26*	0.28*	0.29*	-0.22*	0.25*
Feel like you had a sense of belonging to your local community	0.20*	0.21*	0.19	-0.02	0.33*
Get invited to go out and do things with other people	0.28*	0.32*	0.22*	-0.20*	0.26*
Have friends you could get together with to relax	0.31*	0.34*	0.36*	-0.20*	0.36*
Feel like you were part of a group of friends or community	0.34*	0.31*	0.36*	-0.21*	0.34*
Have someone who understood your problems	0.37*	0.35*	0.34*	-0.31*	0.35*
Have someone you trusted to talk with about your problems and feelings with	0.30*	0.26*	0.39*	-0.30*	0.31*

Correlation coefficients are from Spearman’s correlations

* *p* < 0.05

## Discussion

Little research has been conducted on the health and well-being of adolescent parents. We performed what is, to our knowledge, the first comprehensive analysis of the well-being of teen parents in Washington, DC. The study found that despite frequently cited risks and challenges associated with having children during adolescence, teen parents experience positive health and well-being. This finding helps to create a more comprehensive description of this population and suggests that in addition to potential risks there are also strengths, supports, and positive experiences among teen parents. Teens who had strong social support were more likely to have better perceptions of their health and well-being. This finding indicates that social support may serve as a protective factor for teen parents.

Prior work has shown that teen parents value and benefit from social support [16, 17]. Additionally, the important connection between social support and mental health outcomes among adolescents has been shown in nonparenting adolescents [26]. Our findings add to this literature and show that among teen parents, greater self-reported social support was positively correlated with higher self-rated physical health, mental health, and well-being; positively correlated with experiencing positive emotions; and negatively correlated with experiencing negative emotions. As social support is correlated with superior well-being among teen parents, investing in policies and program design to bolster social supports may improve health and well-being for teen-headed families. Additional study is needed to evaluate the impact of specific types of social support—including family, peer, and community-based—on adolescent parent well-being.

This study also adds to the limited published data about teen parents’ use of community and governmental resources. Although it is considered a “best practice” to refer adolescent parents to programs to mitigate poverty [4, 27], it is unknown if and how teen parents access these resources. In our sample, resources for food access—WIC, SNAP, and food pantries—were the most used, followed by TANF, childcare, and housing supports. Surprisingly, 37% of respondents reported using none of the listed resources. One prior study found higher rates of teen parents accessing governmental and community resources; however, this was a sample of teen parents already engaged in a support program [16]. Our study adds that among a broader citywide sample of teen parents, likely representing varying degrees of engagement with support programs, fewer were accessing resources than expected. Additionally, in our sample, there were no statistically significant differences in health and well-being metrics among those who did and did not use resources. Our study did not assess respondents’ rationale for not using services and did not distinguish between those who had access or knowledge barriers to resource utilization, versus those who chose not to utilize services for other reasons. Future work will examine subgroups of parents who are not accessing resources (for example, fathers) to determine if there are barriers or other reasons that teen parents do not access available resources. We hope that this study also helps to inform future research by highlighting potential strengths and supports of teen parents alongside potential risks. Future research should include qualitative interviews with teen parents to determine the unique contributors to their health and well-being, and to explore further what types of strengths and supports can be bolstered by community interventions.

The study had several limitations. First, data was collected via self-report from the anonymous, online, public survey. To preserve anonymity, we relied only on self-report and did not conduct additional verification as to the identity of survey takers. However, to maximize recruitment of the target population, the survey was advertised through social networks and community programs that focus directly on teen parents in Washington, DC, and responses that were outside of the study inclusion criteria were excluded. Second, this survey may not capture the full experience of all teen parents in Washington, DC. Survey takers had to see the survey link through a program, listserv, or social media account, which implies some degree of connection with programs. There are likely teen parents who are not engaged with these platforms who were missed from the sample. It is also possible that more engaged and healthy teens took the survey. Third, these findings represent the experience of expectant and parenting teens in a single urban city and may not be generalizable beyond Washington, DC.

## Conclusion

This study assessed parenting and pregnant adolescents’ well-being in the domains of health-related factors, social support, and use of governmental and community resources. Despite socioeconomic barriers and stigma faced by teen parents, the respondents reported overall positive physical, mental, and emotional health. Greater social support was correlated with better outcomes in these areas. Additionally, the findings highlighted that a significant number of participants were not connected to governmental and community resources. These results provide a snapshot of the well-being of expectant and parenting teens in Washington, DC. Future work will leverage DC NEXT!’s multidisciplinary, city-wide stakeholder group to translate these findings into policies and programs that meet the needs of this population.

## Data Availability

The datasets generated and/or analysed during the current study are not publicly available due to institutional policies but are available from the corresponding author on reasonable request.

## List of Abbreviations

SNAP: Supplemental Nutrition Assistance Program
TANF: Temporary Assistance for Needy Families
WIC: Special Supplemental Nutrition Program for Women, Infants and Children
